# Editorial: Enhancing salinity tolerance in crops - molecular and practical perspectives

**DOI:** 10.3389/fpls.2024.1367677

**Published:** 2024-02-14

**Authors:** Raj Kumar Gautam, Rakesh Kumar Singh, S.L. Krishnamurthy, Sneh Lata Singla-Pareek

**Affiliations:** ^1^ ICAR-National Bureau of Plant Genetic Resources, New Delhi, India; ^2^ International Center for Biosaline Agriculture, Dubai, United Arab Emirates; ^3^ ICAR-Central Soil Salinity Research Institute, Karnal, Haryana, India; ^4^ International Centre for Genetic Engineering and Biotechnology, New Delhi, India

**Keywords:** salinity, tolerance, haplotypes, halophytes, marker assisted breeding

Presence of excessive salts is a major abiotic stress which adversely affects the agricultural productivity and global food security. Soil salinity is one of the top 10 threats to the world’s land resources, with an annual increase of 1.5 million hectares. A global issue, salinity stress puts ecosystems, food security and productivity at risk. Plant development, physiology, molecular reactions, nutrient and water intake, and seed germination are all impacted by salinity. Anthropogenic activities and growing industrialization exacerbate shortage of cultivable land which necessitates extending crop cultivation to the inhospitable conditions like salt prone areas.

According to recent research, many physiological, biochemical and molecular processes in plants might experience the detrimental effects of salt stress at the tissue and whole plant levels. Advances in genetics and biotechnology have made it possible to breed cultivars that are more tolerant of salt and have higher production potential on soils affected by salt (**Figure 1**). Furthermore, the development of salinity tolerance in plants has been made possible by the discovery of numerous QTLs, genes and transcription factors thanks to advancements in molecular techniques. The Research Topic “*Enhancing Salinity Tolerance in Crops - Molecular and Practical Perspectives*” offers a thorough examination of both original and reviewed research that addresses the various aspects of the role of molecular mechanisms in crop plants’ ability to tolerate salinity in salt-affected ecosystems. We endeavoured to invite, review and compile original and review papers in this section.

**Figure 1:**
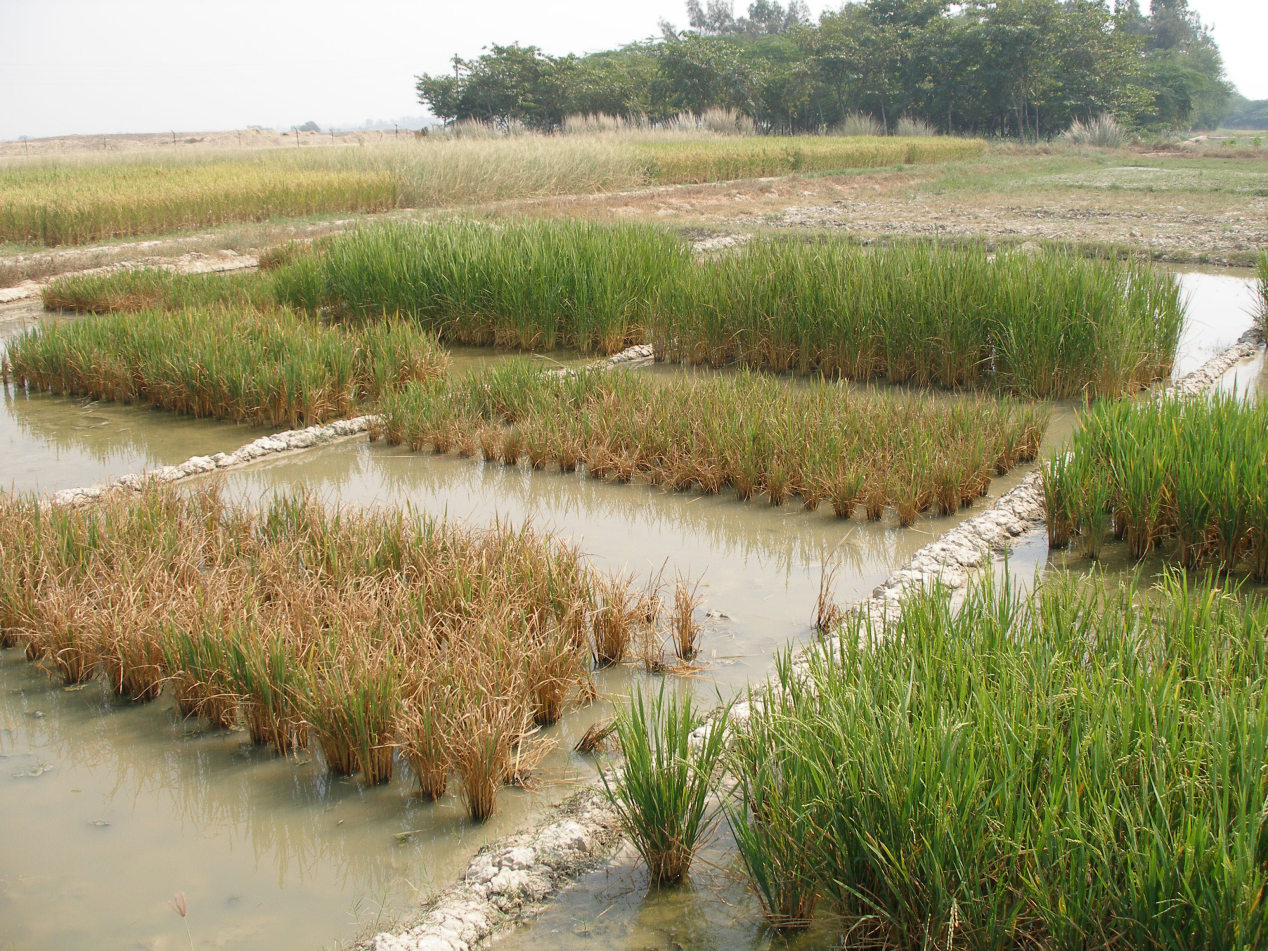
Phenotypic performance of tolerant and sensitive rice genotypes in natural sodic soil stress.

*Gossypium hirsutum* and *G. darwinii* seedling stages were used to identify salt stress-tolerant candidate genes in the BC2F2 population using NGS-based bulked segregant analysis (Shehzad et al.). Six functional SNPs were found in four genomic locations which were connected to five potential genes. *Gohir.D05G367800* and *Gohir.D12G23910*, two up regulated genes in the salt-tolerant species *G. darwinii*, were further validated among these five candidate genes. The two salt-tolerant cotton genes, *Gohir.D05G367800* and *Gohir.D12G239100* require more validation in transgenic and CRISPER CAS cotton technology for their use in development of salt tolerant cotton varieties.

Sequence differences were found in the *ORF, 1-kb 3′UTR, 2-kb 5′UTR*, and the other five salt-tolerant-related genes (Li et al.). For every gene (*SKC1/OsHKT8, GS3.1, OsHAK21.1, OsPAO3* and *RST1/OsARF18*), a haplotype analysis was performed. Strong intragenic markers were created for genes relevant to salt tolerance and 24 representative rice accessions were used to assess the markers’ efficacy. Six SNPs and two Indels in the 5′UTR, five SNPs on exons, two SNPs on intron 2, and two SNPs in the 3′UTR were among the distinctive variants found in the elite group HapA and HapB of gene RST1/OsARF18. Two CAPS indicators that have been developed - “ARF18 5UC” and “ARF18 E2C” for salt tolerance may be further confirmed using gene editing. Pyramiding of these elite haplotypes could be further used in the breeding programme to improve salt tolerance of rice cultivars.

Salinity prone environments are often affected by bacterial blight (*Xanthomonas oryzae* pv*. oryzae*) disease of rice. Marker-assisted pyramiding enhanced bacterial blight (BB) resistance in a salt-tolerant rice variety for sustaining rice production of tropical islands (Gautam et al.). In a coastal saline tolerant variety CARI Dhan 5, pyramiding resistance genes (*Xa4, xa5, xa13* and *Xa21*) by marker-assisted backcross breeding (MABB) was accomplished. In addition to possessing salinity tolerance, CARI Dhan 5 x IRBB60 derivatives possessing combinations of *Xa/xa* genes exhibited BB resistance across locations and years. The environment-friendly rice farming system in the geographically isolated Andaman and Nicobar Islands is anticipated to benefit greatly from these genetically upgraded salt tolerant and BB resistant CARI Dhan 5 lines.

Rice production is increasingly being hampered by the combination of drought and salinity stress, particularly in river deltas and coastal regions where low rainfall not only lowers soil moisture levels but also decreases river water flow, allowing saline seawater to seep in. Development of a phenotyping protocol for combined drought and salinity stress at seedling stage in rice is very timely and useful (Kota et al.). The optimal approach applied both pressures at the same time by planting rice at 75% of field capacity in saline soil, allowing the rice to gradually dry down. Meanwhile, when drought stress was administered at the vegetative stage alone, physiological characterization showed that chlorophyll fluorescence during the seedling stage associated well with grain output. In order to generate dual-stress tolerant rice varieties with improved resistance to combined stressors, rice breeding populations can be screened using the developed drought + salinity protocol.

In a review paper, halophytes are commonly described as plants that can thrive well and complete their life cycle in extremely salinized conditions with at least 200–500 mM of salt solution (Mann et al.). During the last few decades, a range of salt-tolerant grasses and halophytes has been studied in order to extract salt-tolerant genes and evaluate how well they team up together to augment crop plants’ ability to cope up with excessive salts. However, the absence of complete genetic information and the unavailability of any model halophytic plant system limits the usefulness of halophytes. Even though the majority of salt tolerance studies currently employ Arabidopsis (*Arabidopsis thaliana*) and salt cress (*Thellungiella halophila*) as model plants, these plants are short-lived and have a limited ability to withstand salinity. The realm of halophytes could serve as reservoir for unravelling the molecular mechanisms and their genetic introgression for developing robust salt tolerant cultivars through modern technologies.

To sum up, this Research Topic on improving salinity tolerance in crops through both molecular and practical perspectives incorporates a broad range of original studies, review and meta-analyses that address most, if not all, facets of the present advancements in this domain. It is hoped that researchers would recognize the wide range of facets involved in the Research Topic and be inspired to explore novel avenues of inquiry that will place plants at the center of crop plants’ ability to withstand salts. Alleviating the salt stress factors through engineering the innate mechanisms of crop plants would be a fantastic way to accomplish the Sustainable Development Goals and improve the production potential in salt-affected ecosystems in the era of climate change.

## Author contributions

RG: Conceptualization, Data curation, Formal analysis, Investigation, Methodology, Project administration, Resources, Supervision, Validation, Visualization, Writing – original draft, Writing – review & editing. RS: Writing – original draft, Writing – review & editing. SK: Writing – original draft, Writing – review & editing. SS: Writing – original draft, Writing – review & editing.

